# Step-by-Step Esthetic Rehabilitation with Chairside System

**DOI:** 10.1155/2021/5558158

**Published:** 2021-03-25

**Authors:** Rim Kallala, Mohamed Habib Chaouch, Karim Nasr, Teva Courset

**Affiliations:** ^1^Dental Faculty of Monastir, Tunisia; ^2^University of Monastir, Research Laboratory of Occlusodontics and Ceramic Prostheses LR16ES15, 5000 Monastir, Tunisia; ^3^Department of Dental Faculty at the Dental Faculty of Monastir, Tunisia; ^4^Faculty of Dental Surgery and University Hospital, Toulouse, France

## Abstract

In modern dentistry, Computer-Aided Design and Manufacturing (CAD/CAM) is a promising technology that allows fabrication of prosthetic restorations through milling procedures. Over years, with the continuous improvement of technology, direct CAD/CAM or “chairside” technology is becoming a widespread approach which offers immediate rehabilitation with long-term rates reported by several studies compared to conventional techniques. All steps are generally carried out in the dental office during the same treatment session. The present paper is about a healthy female patient with a decayed 36 tooth which was restored by ceramic onlay using Planmeca's PlanCAD system. Through the present clinical case, a detailed protocol of chairside technology would be presented from the digital impression to the milling process. It would detail impression steps. It would also highlight especially the virtual design confection of prosthetic restoration using a biogeneric model included in the software. It also illustrated tools which could be used by the dentist to perform the design. Also, some useful tips would be presented in order to perform the confection. On this subject, various studies showed the viability of such technology. To summarize, referring to previous studies, this promising technology allows especially time-saving and patient's comfort compared to the indirect one.

## 1. Introduction

In modern dentistry, Computer-Aided Design and Manufacturing (CAD/CAM) is a promising technology that allows fabrication of the prosthetic restorations through milling procedures [1]. Over years, with the continuous improvement of technology, direct CAD/CAM or “chairside” technology is becoming a widespread approach which offers immediate rehabilitation. The first one was introduced by the CEREC system [1, 2]. Due to this technology, all steps are generally carried out in the dental office during the same treatment session. CAD/CAM systems registered a constantly increasing use in many fields of dentistry and allow a completely digital workflow, from impression to the final framework, with good clinical reliability [2, 3] and excellent patient feedback [4]. The purpose of this paper was to detail the protocol of chairside system and provide a useful evaluation tool, through a clinical case carried out in the dental clinic of the Faculty of Dental Surgery Paul Sabatier Toulouse. The direct CAD/CAM system used was Planmeca's PlanCAD.

## 2. Clinical Presentation

**Figure 1:**
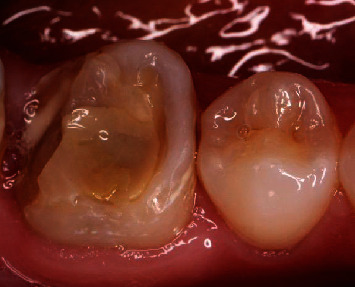
Initial situation.

It was a 34-year healthy female patient who consulted for rehabilitation of the left first lower molar (Figure 1). She had high esthetic expectations. The clinical examination showed good oral hygiene and a decayed and nonvital 36 tooth. Radiological examination confirmed a good quality of root canal filling.

**Figure 2:**
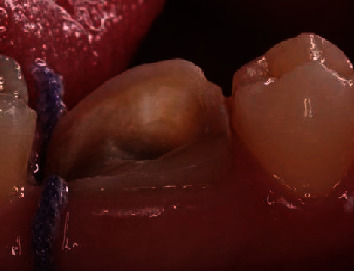
After preparation with placed retractor cord before impression.

**Figure 3:**
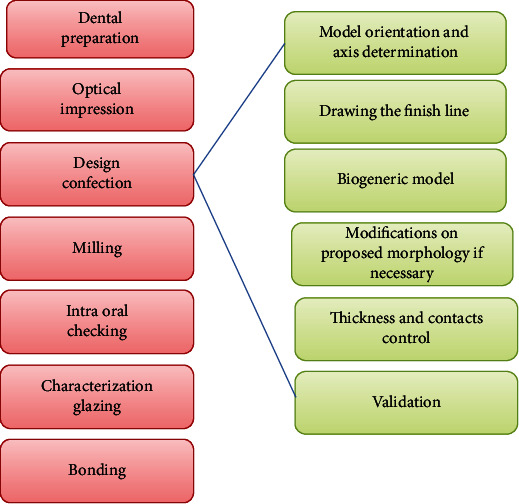
Chairside workflow.

In accordance with the therapeutic gradient and taking into consideration residual dental tissue, it was decided to place a ceramic onlay using the direct CAD/CAM technique. The first step was the removal of decayed tissue. Dictated by caries morphology, preparation was done while preserving the maximum of enamel tissue and avoiding sharp angles and wide shoulders. After preparation, dental residual tissue was mainly supragingival which would facilitate optical impression and future bonding (Figure 2). All the workflow is summarized in Figure 3.

### 2.1. Computer-Aided Design and Manufacturing Steps

#### 2.1.1. Administrative File (Figure 4)

At the software level (Romexis, Planmeca Oy 4.0, Helsinki, Finland), the concerned tooth, restoration type, and material were selected.

#### 2.1.2. Digital Impression (Figure 5)

Before the digital impression, a retraction cord (Pro Retract, FGM, Jovinile Brasil) was placed in the sulcus and the tooth was air-dried. Then, intraoral scanning was carefully performed using the Planmeca Emerald camera which is a powder-free technology. It was a true-color high-resolution procedure. Three sectoral impressions were necessary: concerned and antagonist sectors (intraoral scan), as well as recording of the occlusion (buccal scan). At this stage, it was possible to switch the models to the monochrome mode in order to visualize the accuracy of the recording. Likewise, it was easy to check the available prosthetic thicknesses and whether there are any gaps in the impression. If necessary, the impression could be retouched and completed. Also, unnecessary areas could be cleaned using an eraser tool.

#### 2.1.3. Design Confection of the Prosthetic Restoration Figures 6 and 7

The virtual cast orientation was the first step. Determination of the insertion path and the model axis was then necessary. After that, the finishing line was rigorously drawn. This operation was semiautomatic and should be controlled by the practitioner. The software, thus, after analyzing the neighboring teeth, proposed a design of the future restoration. It could be modified through variable tools: displacement, fine retouching, and adjustments of the contact zones (occlusal and proximal). The color code indicated the intensity of these contact zones. An analysis of the material thicknesses could also be done in order to avoid areas of extreme thinness which may cause fractures. Before the milling process, the virtual onlay would be visualized.

#### 2.1.4. Manufacturing or Milling Process

Leucite-reinforced glass ceramics was chosen for the present case. The operator, then, inserted the appropriate ingot (material and size) inside the machine. Via wireless, data were sent to the computer-controlled milling unit. The milling machine (PlanMill 40S, Planmeca, Helsinki, Finland) uses dual spindles and could mill simultaneously two sides of restoration according to customized and calculated milling paths. Finally, the milling process was rapidly performed and took 12 min. At the end of machining, the block was removed from the machine and then separated from the machining lug.

After intraoral checking, only minor adjustments were done using diamond instruments. Modified zones were then polished. The restoration underwent a finishing phase (coloring, glazing, and polishing) before the bonding.

**Figure 8:**
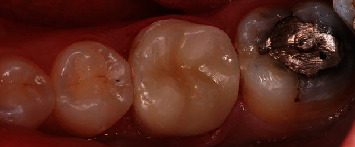
Final outcome after bonding.

The patient was happy with the final result (Figure 8).

## 3. Discussion

Today, chairside or direct CAD/CAM systems are a promising technology saving time with total independence from the laboratory technician and better communication with the patient [4–6]. The indirect CAD/CAM systems or classical methods usually require conventional impression [1, 7]. On obtained gypsum casts, digital acquisition would be done and the milling process would be performed in the dental laboratory [7–12]. The major flaw of conventional impression is the risk of errors due to material dependence especially the dimensional deformations during polymerization. According to Boitelle and Fromentin [13, 14], there is a physical dispersion which could drive to errors and spoil the precision and the quality of the dentoprosthetic joint. Lima et al. [15] reported statistically significant differences by comparing the marginal adaptation of prosthetic restorations milled through both direct and indirect CAD/CAM systems in favor of the direct one. The study of Carvalho et al. [16] confirmed the superiority of digital techniques in comparison with conventional methods. However, the systematic review of Goujat et al. [17] reported that the marginal adaptation obtained was satisfactory without statistically significant differences between the two techniques. The meta-analysis could not be performed as findings were heterogeneous. For Aswani et al. [18], variable results were reported depending on the systems used. According to Ender and Mehl [19], optical impressions provided levels of accuracy in the same order as those of conventional impressions. Some studies [19–21] reported statistically not significant differences between the impression techniques. Indeed, Boitelle and Fromentin [13] reported that physical dispersion exists also for the direct CAD/CAM technique. It could be linked to different interactions of the light beam and dental tissues. Besides, errors could be due to the oral environment, to external environments (brightness of the treatment room), and to the manipulation also [13]. The study of Kuhr et al. [21] pointed out that conventional casts seem to be similar to digital impression models. But secondary areas, such as grooves and pits, were better reproduced in gypsum models. To sum up, chairside technology allows especially saving time [16] as digital scanning is easy and rapid. Besides, there is no need to disinfect and clean dental impressions either waiting cast pouring.

On the other hand, the restoration occlusal design is considered among the most important conditions for an optimal outcome, through harmonic relation to adjacent teeth and interference-free occlusal contacts [22]. In CAD/CAM technology, it is adjusted thanks to an algorithmic equation which involves a library of intact tooth morphology integrated into the software called the biogeneric model [6, 23]. In this regard, several studies have been carried out comparing the morphologies of natural teeth and those given by the biogeneric models and have concluded with similar morphologies [7, 24, 25]. Ender et al. concluded that according to experts, obtained morphologies were natural looking [24]. Others compared the morphology of natural teeth and those of wax-ups made by laboratory technicians in favor of the biogeneric model [26, 27].

Otherwise, in the present clinical situation, the tooth could be reconstructed with a fiber post and core followed by a crown.

Nevertheless, this promising technology has some limitations: The first is the high cost which requires huge investment. Then, as it provides monolithic restorations, the esthetic outcome would be better using a stratification technique which allows characterization in the framework depending on the laboratory technician's skills and performances [26]. Tuncel reported statistically significant differences in terms of translucency between monolithic zirconia and framework zirconia [29].

## 4. Conclusion

The arrival of CAD/CAM technology, particularly the chairside system, has revolutionized dental prostheses. It is a promising technique which allowed especially time-saving and comfort for the patient. The appropriate use of corresponding software, as well as the adjustment method, is required for successful restorations. Also, an appropriate occlusal context and adequate bonding, respecting the properties of each material, would guarantee the longevity of the future restoration.

## Figures and Tables

**Figure 4:**
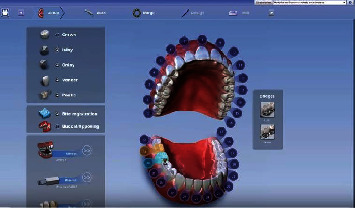
The administrative file.

**Figure 5:**
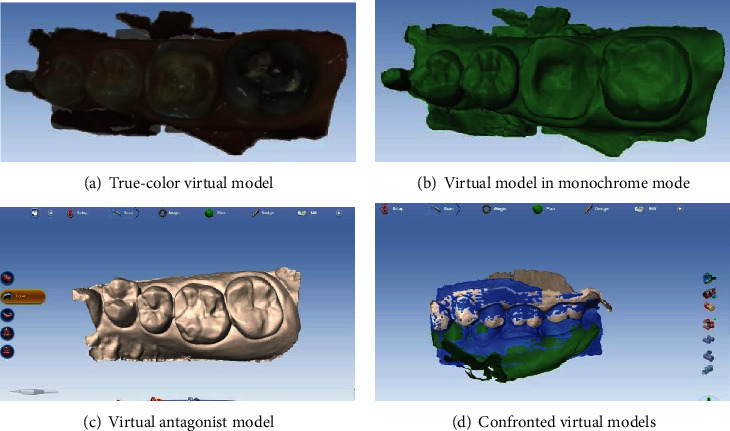
Virtual models obtained after digital impression.

**Figure 6:**
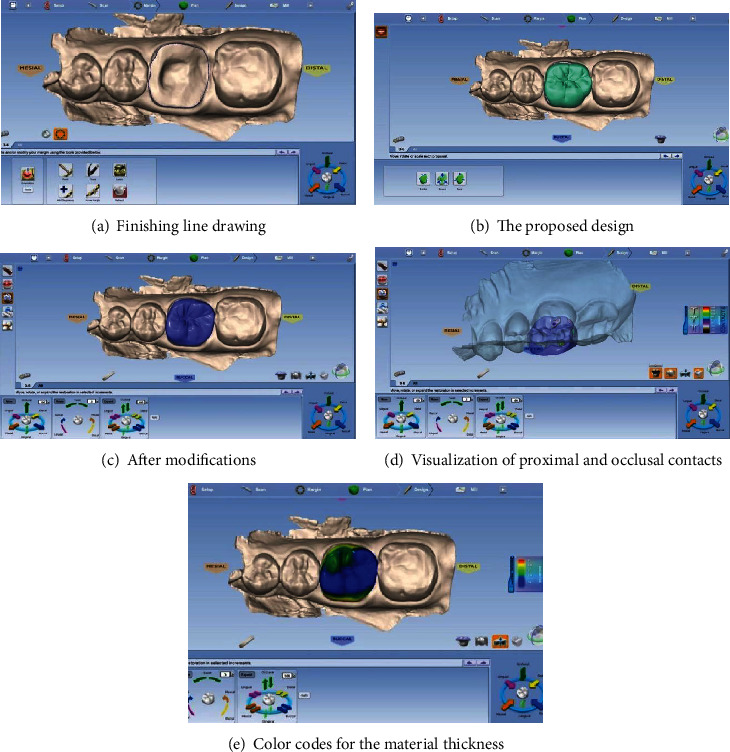
Design confection of the future restoration.

**Figure 7:**
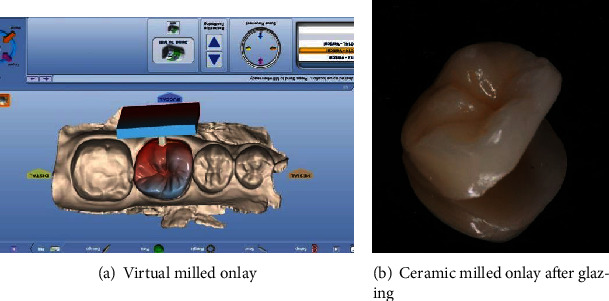
Milled onlay.
